# Multidimensional Measures of Impulsivity in Obsessive-Compulsive Disorder: Cannot Wait and Stop

**DOI:** 10.1371/journal.pone.0111739

**Published:** 2014-11-05

**Authors:** Sung Yun Sohn, Jee In Kang, Kee Namkoong, Se Joo Kim

**Affiliations:** Department of Psychiatry and Institute of Behavioral Science in Medicine, Yonsei University College of Medicine, Seoul, Korea; Bellvitge Biomedical Research Institute-IDIBELL, Spain

## Abstract

**Objective:**

Although the relationship between obsessive compulsive disorder (OCD) and impulsivity has long been debated, impulsivity has not been systematically examined in clinical samples of OCD. Meanwhile, recent findings suggest that impulsivity is multi-dimensional construct that can be examined through several constructs. Therefore, this study is aimed to evaluate multiple facets of impulsivity in OCD.

**Method:**

The recruitment includes 80 OCD and 76 healthy control participants. Participants completed a test battery comprising three behavioral tasks of stop signal task (SST), delay discounting task (DDT) and balloon analog risk test (BART), and one self-report measure of the Barratt Impulsiveness scale (BIS-11).

**Results:**

OCD subjects showed significantly lower stop signal reaction time of SST reflecting higher action impulsivity and higher delay discounting parameter of DDT suggesting increased choice impulsivity but significantly lower adjusted mean pump of BART implying lower risk taking propensity of BART than healthy control.

**Conclusion:**

Increased Action and choice impulsivity, and decreased risk taking propensities were found in OCD. These findings seem to be consistent with clinical characteristics of OCD such as greater preference for or avoid risky situations (avoidance), inability to wait tension relief may provoke safety behaviors (compulsion) and inability to stop already started behaviors (repetition).

## Introduction

Obsessive-compulsive disorder (OCD) is a common psychiatric condition characterized by obsessions and compulsions. *Obsessions* are repetitive, unwanted, intrusive thoughts, images, or impulses causing uneasiness, apprehension, or distress in one's mind. *Compulsion* is repetitive ritualistic behavior and is defined as actions inappropriate to the situation that nevertheless persist and which often result in undesirable consequences [1].

Like compulsivity, impulsivity is a common feature in various psychiatric disorders. Impulsivity involves actions that are insufficiently conceived, prematurely expressed, excessively risky or inappropriate to the situation, and that often lead to undesirable outcomes [1]. According to the traditional conception, compulsive disorders and impulsive disorders represent opposite ends of a single dimension, with the former on harm avoidant and the latter on risk seeking [2], [3]. However, recent research suggest that, rather than being polar opposites, compulsivity and impulsivity may represent orthogonal factors that each contribute in varying degrees to various psychiatric conditions, including obsessive-compulsive spectrum disorders (OCSDs) [4]. Phenomenologically, OCSDs are characterized by difficulties suppressing repetitive behaviors that are inappropriate to the situation, suggesting underlying impairment in inhibitory control [5]. In the fifth edition of the Diagnostic and Statistical Manual of Mental Disorders (DSM-5), OCD has been reclassified within a new chapter of obsessive-compulsive and related disorders (OCRDs) that includes trichotillomania and skin picking, in which impulsive features are core characteristics [6]. In some aspects, both impulsive and compulsive disorders show similar clinical features, such as difficulties in delaying or inhibiting repetitive behaviors [7]. Compulsive and impulsive disorders are often comorbid and influence each other's development. A number of studies reported high prevalence of impulse control disorders (ICDs) in OCD [8] and high prevalence of OCD in ICDs [9]. In addition, the impulsiveness in OCD seems to have various significant clinical implications. Comorbid ICDs in patients with OCD are associated with poor clinical characteristics, such as early age at onset, great number and severity of symptoms, poor insight, insidious onset, impaired functioning, and poor treatment response seen at long-term follow-up [10]. OCD subjects with higher impulsivity show higher learning problems, low frustration tolerance, poor interpersonal relationships, attention-seeking behavior in childhood, higher neuroticism, and a higher incidence of somatic symptoms [11]. Additionally, based on neuroimaging and lesion studies, one of the major areas involving impulsivity is the ventral striatal loop [12], which is a target area of deep brain stimulation to improve obsessive-compulsive (OC) symptoms in refractory [13]. Despite this substantial evidence suggesting the importance of impulsivity in OCD, there have been few studies on this relationship and these have mainly used self-rating measures [3], [14].

Impulsivity is not a unidimensional construct and it has been suggested that there are several distinct facets of impulsivity, including behavioral disinhibition (impulsive action), impulsive decision making (impulsive choice), and unduly risk taking [15], [16], [17]. Behavioral disinhibition is defined as an active process that involves suppression of a prepotent response that has been actively investigated by using the stop signal task (SST) [15]. Impulsive decision making is characterized by making choices for smaller immediate rewards rather than waiting for larger delayed rewards. The delay discounting task (DDT) is a well-known behavioral task that measures delay discounting, which refers to the devaluing of a reward due to its location in the future; in other words, DDT assesses the tendency to discount future rewards [18]. Risky decision making is the tendency to engage in behaviors with some potential for danger or harm while also providing an opportunity to obtain some form of reward [19]. The balloon analogue risk task (BART) is a computerized measure that assesses the tendency to engage in simulated risk taking behavior in a context in which unduly risky behavior results in poor outcomes [17]. Considering the multidimensionality of impulsivity, it would be fruitful to simultaneously evaluate the various dimensions of impulsivity (impulsive choice, impulsive action, and risk taking) as well as by using a self-rating measure. These approaches may help in providing a more integrative understanding about the various subtypes of impulsivity and their interrelations in OCD.

Therefore, the central aim of this study is to explore how subconstructs of impulsivity pertain to OCD. We systematically assessed impulsivity using both a subjective self-report and an objective behavioral approach with multiple measures. Based on fact that avoidance behavior and difficulties of inhibiting or delaying compulsive urges are characteristic features of OCD, we hypothesized that OCD subjects show higher impulsive actions and choices and make lower risky decisions than normal controls.

## Materials and Methods

### Participants

The study group comprised 80 patients with OCD and 76 healthy control participants who were matched for age and sex. The primary diagnosis of OCD and other comorbid psychiatric conditions in patients were determined by the patient version of the Structured Clinical Interview for the DSM-IV (SCID-IV) [20], assessed by a trained psychiatrist (S. J. Kim). Healthy control subjects also underwent SCID-IV and were required to be free of a lifetime history of psychiatric illness.

Exclusion criteria for OCD patients demanded the absence of significant medical or neurologic illness and any other Axis I disorders except for comorbid major depressive disorder. All participants gave written informed consent according to the procedures approved by the Severance Hospital Institutional Review Board.

### Procedure and clinical assessments

To assess OCD symptoms, we administered the Yale-Brown Obsessive-Compulsive Scale (Y-BOCS) [21] and the Obsessive-Compulsive Inventory Revised-Korean, (OCI-R-K) [22]. Depressive symptoms were assessed using the Montgomery-Åsberg Depression Rating Scale (MADRS) [23]. In addition, the vocabulary and block design of the Korean version of the Wechsler Adult Intelligence Scale was applied to all participants to estimate IQ. The participants were excluded if their IQ was below average (IQ <90) [24], as some studies had suggested associations between intelligence and the performances of SST [25], DDT [26], and BART [27].

### Action impulsivity: Stop Signal Task (SST) [28]

Response inhibition (action impulsivity) was assessed using the SST, which consists of 120 total trials. In each trial, participants were presented with the *go* stimulus (the letter “X” or “O”) for 1,000 ms with participants instructed to press the Z key for an X and the/key for an O as quickly and as accurately as possible (go trials). For stop trials (25% of trials), a go stimulus was followed by a stop signal (a beep) after a variable delay, which signaled participants to withhold a response. The onset of the stop signal was varied by a tracking algorithm, in which the stop signal delay was initially 250 ms and was decreased by 50 ms after a previous stop task failure and increased by 50 ms after a previous success. To yield reliable stop signal reaction time (SSRT), we used outlier criteria as follows: (1) percent inhibition on stop trials less than 25% or greater than 75%, (2) percent go-response less than 60%, (3) percent go-errors greater than 10%, and (4) SSRT estimate that is negative or less than 50 ms [29]. A stop signal delay (SSD) indexed the time delay the participant needed in order to inhibit their response. Go reaction time (GORT) is the time taken to press the button when there is no auditory signal. The main dependent variable, SSRT, is a sensitive measure of response inhibition and was extracted by the quantile method which does not require an assumption of 50% inhibition [28]. Longer SSRT reflects worse inhibitory control (slower inhibitory process). In this study, we used the Korean version of the SST [30].

### Choice impulsivity: Delay Discounting Task (DDT) [31]

Delay discounting (choice impulsivity) was assessed using a binary choice procedure. In each trial, the computer screen showed a series of choices between two virtual money rewards: immediate smaller reward and delayed larger reward. The delayed reward was fixed at 1,000,000 Korean Won (???), which is approximately 100 US dollars. At the first session, the amount of delay was held constant as within 1 week, and the 26 immediate rewards were presented on the screen in descending order, one per each trial. In the next session, the sequence of immediate monetary rewards ascended in amount until the largest reward was presented, with a particular temporal delay. The next sessions were repeated with incrementally larger temporal delays of 1 week, 2 weeks, 1 month, 6 months, 1 year, 3 years, and 10 years. Within each session, the amount of the immediate monetary value that was preferred equivalently to the large delayed monetary value was defined as the point of subjective equivalence (i.e., an indifference point). Indifference points across the delays were calculated using the hyperbolic decay function, yielding k values reflecting the delay discounting rate [32]. Higher k values indicate higher sensitivity to delayed rewards or choice impulsivity. In this study, we used the Korean version of the DDT [33].

### Risk Taking: Balloon Analogue Risk Task (BART) [34]

During the BART, participants were required to press a button to inflate a series of 30 balloons. With each button click the balloon inflated and participants earned money (50 ???) for each pump. This money was added in a temporary bank for that balloon. Participants were told that at some point the balloon would pop and they would lose all the money in the temporary bank. When a balloon exploded, an explosion sound and a picture of the exploding balloon were generated by the computer. Participants were instructed that they could collect from the temporary account to their permanent account at any point before the balloon exploded by pressing a button marked “Collect.” After each time a participant collected or popped a balloon, a new balloon appeared. Participants did not actually receive the money, but were instructed to imagine that they would have earned money in a permanent bank in real life. Risk taking propensity was measured by calculating the adjusted mean pumps (AMP), the average number of inflations over the trials in which the balloons did not explode. A larger adjusted value represents a higher risk taking propensity. As the Korean version of the BART was not available, we used the original version of BART [34], which was translated into Korean.

### Self-report impulsivity questionnaire: Barratt Impulsiveness Scale (BIS) [35]

The BIS-11 is a self-rating questionnaire. The scale consists of three factors of impulsivity: (1) motor impulsiveness, (2) attentional (cognitive) impulsiveness, and (3) non-planning impulsiveness. In this study, the Korean version of the BIS-11 [36] was used.

### Statistical analyses

Statistical tests were two-tailed with level of significance set at *p* = 0.05. Demographic characteristics (age, estimated IQ) were compared across groups using the Mann-Whitney U test or *t*-test for continuous variables. Gender was compared using χ^2^ analysis. The primary analyses were *t*-tests with k parameters, adjusted value, SSRT, BIS total score, and BIS subscale as the dependent variables. Because the k parameters were not normally distributed, the distributions of the k parameters were normalized by using the natural log transformation. In addition, we explored associations between the task parameters, BIS scores, and OCD symptom dimensions in patients using Pearson's correlation analyses. All analyses were performed using SPSS version 20.0 (SPSS Inc., USA).

## Results

### Sample characteristics

Demographic and clinical characteristics for participants are presented in Table 1. A total of 80 OCD patients underwent testing. There were no significant differences in age, sex, or estimated IQ between OCD patients and healthy controls. Patients with OCD presented in the moderately ill range. The mean age at onset of OCD was 18.9±9.3 years. In the OCD groups (n = 80), 78 were currently taking psychiatric medications, all were taking serotonin reuptake inhibitors (SRIs), 31 were taking an SRI with a non-SRI (i.e., benzodiazepine, n = 24; benzodiazepine and antipsychotic, n = 7).

**Table 1:** Demographic and Clinical Characteristics of OCD and Healthy Control Subjects.

	OCD (n = 80)		HC (n = 76)		U/χ2/t	*p*
	Mean	SD	Mean	SD		
**Age (Years)^a^**	27.8	8.1	25.5	4.1	2797	0.39
**Male (%)^b^**	73.8		65.8		1.17	0.28
**IQ Estimate^c^**	111.7	10.8	112.0	10.5	−0.176	0.86
**Y-BOCS**	21.7	6.9				
**MADRS**	16.4	9.7				
**OCI-R-Total Score**	34.6	14.2				
** Washing**	5.3	3.8				
** Obsessing**	7.8	2.8				
** Checking**	6.0	3.4				
** Ordering**	5.1	3.6				
** Hoarding**	4.4	3.6				
** Neutralizing**	6.1	3.7				

HC, healthy control subjects; OCD, Obsessive-Compulsive disorder; Y-BOCS, Yale-Brown Obsessive Compulsive Scale; MADRS: Montgomery-Asberg Depression Rating Scale; OCI-R, Obsessive Compulsive Inventory-Revised.

a. Mann-Whitney U test because the data were not normally distributed.

b. χ2-test.

c. Independent samples two tailed t-test.

### Group comparisons

Behavioral performance and self-report impulsivity are presented in Table 2. Results on the SST were analyzed after first excluding data from 20 OCD patients and 24 normal controls by the outlier criteria method described in [29].

**Table 2:** Comparison of impulsiveness in patients with OCD and healthy controls.

	OCD (n = 80)		HC (n = 76)		t	*p*	*Cohen's d*
	Mean	SD	Mean	SD			
**BART**							
AMP	27.75	18.25	34.64	16.51	2.469	0.015*	0.40
**DDT**							
log k	−4.04	2.50	−5.09	1.81	−3.010	0.003**	0.48
**BIS**							
BIS_A	16.65	2.73	14.36	2.27	−5.725	<0.001**	0.91
BIS_M	17.14	3.96	14.36	3.50	−4.639	<0.001**	0.74
BIS_NP	22.41	4.30	21.43	3.29	−1.589	0.114	0.26
BIS_T	56.20	9.21	50.14	7.34	−4.553	<0.001**	0.73
**SST (OCD, n = 60; HC, n = 52)**							
GORT	518.83	128.28	522.31	112.77	0.151	0.880	0.03
SSD	353.17	106.34	373.85	104.80	1.034	0.304	0.20
SSRT	165.67	48.94	148.46	35.83	−2.141	0.035*	0.40
STOPACC	0.62	0.10	0.61	0.13	0.711	0.479	0.09

BART, Balloon Analogue Risk Task; AMP, Adjusted Mean Pumps; DDT, Delay Discounting Task; BIS, Barratt Impulsiveness Scale; BIS_A, Barratt Impulsiveness Scale – Attentional; BIS_NP, Barratt Impulsiveness Scale – Non-planning; BIS_M, Barratt Impulsiveness Scale – Motor; BIS_T, Barratt Impulsiveness Scale – Total; SST, Stop Signal Task; GORT, Go Reaction Time; SSD, Stop Signal Delay; SSRT, Stop Signal Reaction Time; STOPACC, stop accuracy.

* p<.05, significant difference. ** p<.01, significant difference.

OCD participants had significantly longer SSRTs than did those in the control group (*p* = 0.04). No significant differences between the groups were found on GORT, and there were no impairments in responding (*p* = 0.88). OCD participants exhibited significantly higher AMP than did controls (*p* = 0.01). The mean log k was significantly higher in the OCD group than in the healthy control group (*p* = 0.005). On the BIS, the OCD group evidenced greater levels of total impulsivity than the healthy controls (*p*<0.001). OCD patients reported significantly higher levels of attentional impulsivity and motor impulsivity than did those in the control group (all *p*<0.001). There were no significant group differences in non-planning impulsivity.

### Correlations between measures of impulsivity

Correlational analyses among clinical variables and impulsivity measures were performed for OCD patients. The results are presented in Table 3. The severities of obsessive-compulsive symptoms (Y-BOCS) and depressive symptoms (MADRS) were not significantly correlated with any task measures (all *p*>0.05). In addition, the three behavioral measures (SSRT, DDT, and BART) were not significantly correlated with each other (all *p*>0.05). The correlations between the behavioral measures and the BIS also were not significant (all *p*>0.05). We obtained the same results when we performed correlational analyses for the controls and for the combination of both OCD and control participants (data not presented).

**Table 3:** Pearson Correlation matrix (n = 80) comparing all measures of impulsivity in patients with OCD.

	1	2	3^a^	4	5	6	7
**1 AMP**	-	−0.127	0.054	0.161	0.133	0.156	0.178
**2 log k**		-	0.191	0.083	−0.214	0.005	−0.070
**3 SSRT^a^**			-	−0.048	−0.041	−0.018	−0.041
**4 BIS_A**				-	0.404**	0.462**	0.686**
**5 BIS_M**					-	0.692**	0.873**
**6 BIS_NP**						-	0.902**
**7 BIS_T**							-

AMP, Adjusted Mean Pumps; SSRT, Stop Signal Reaction Time; BIS_A, Barratt Impulsiveness Scale – Attentional; BIS_NP, Barratt Impulsiveness Scale – Non-planning; BIS_M, Barratt Impulsiveness Scale – Motor; BIS_T, Barratt Impulsiveness Scale – Total.

a. analysis was performed with 60 OCD patients remained after excluded by outlier criteria of SST.

*p<.05, significant difference ** p<.01, significant difference.

## Discussion

The main aim of this study was to investigate the relationship between impulsivity and OCD. To the best of our knowledge, this is the first study using behavioral measures of multiple facets of impulsivity (action impulsivity, choice impulsivity, and risk taking) in OCD. Compared to controls, OCD patients showed higher action and choice impulsivity but lower risk taking propensity. On the self-report questionnaire, BIS-11, OCD participants showed higher scores on attentional and motor but not non-planning impulsivity subscales than did controls.

On the SST, OCD participants showed slower SSRTs than controls did, which indicates that more time was required for OCD patients to inhibit a response and which reflects difficulties with motor inhibitory control. Consistent with this finding, several previous studies also reported impaired response inhibition in OCD [5], [37], [38], [39], [40]. Moreover, greater SSRT has also been found in first-degree relatives of those with OCD that did not differ from that of OCD patients [5], [41]. These findings suggest the possibility of impaired motor inhibition for the endophenotype of OCD. In an imaging study, behavioral impairment indicated by the SST was significantly associated with decreased grey matter in orbitofrontal regions and increased grey matter in cingulate, parietal, and striatal regions [42], [43], which have been considered to be implicated in the pathophysiology of OCD [42]. A recent study using non-clinical samples of participants with high SSRT showed increased uncertainty and memory distrust as a consequence of repeated checking compared to participants with lower SSRT [44]. The authors suggested that longer SSRT reflecting poor inhibitory control might increase the liability for harmful effects of neutral compulsive-like behaviors, and the inhibitory deficit might contribute to the development and maintenance of OCD, especially compulsive behaviors [44]. Because the go cues always precede the stop signal in the SST, the increased SSRT of OCD patients indicates that OCD patients fail to inhibit already started actions [45]. These findings are consistent with clinical manifestations because OCD subjects generally are not able to inhibit their compulsive behaviors even though they know them to be senseless, and moreover, once compulsive behaviors begin, usually they become more and more severe.

In the current study, the delay discounting parameter measured by the DDT was higher in the participants with OCD than in the controls. This result suggests that people with OCD tend to choose immediate smaller rewards over larger but postponed ones. This result is in consistent with the clinical characteristics of OCD because most OCD subjects cannot stop or delay their urge to do compulsive behaviors that immediately reduce their tension, even though they result in more negative consequences in the future. To date, there has been only one study of the association between delay discounting and OCD. Pinto et al. [46] examined the asymmetric discounting (intertemporal choice) task in 25 participants with OCD, 25 with OC personality disorders (OCPD), 25 with comorbid OCD + OCPD, and 25 healthy controls. Contrary to our study, they did not find any differences of performance between OCD and controls, although individuals with OCPD show less temporal discounting than did the controls or those with OCD [46]. Although the reasons for the discrepant results are not clear, there were several differences between the two studies. Pinto et al. [46] used a single delay (i.e., 3 months) and discount factor (δ) as a discount parameter, but we used multiple time delays and k parameter, which have been most widely used for delay discounting. In addition, they focused on OCPD rather than OCD and analyzed OCD and OCPD separately, whereas we did not consider the comorbidity of OCPD. Finally, there might be cultural differences between the OCD subjects participating in the two studies. In our study, the participants were all Korean, but in their study, the participants were recruited from one anxiety clinic in North America, and the ethnicities were not presented. However, there has been some evidence suggesting cultural influences on the performance of delay discounting [34]. One recent study reported cultural differences of neural substrates of delay discounting [47]. In addition, the cultural backgrounds may lead to some differences of attitudes toward delay, the perception of time, or the perceived magnitude of the monetary outcome, all of which can influence the delay discounting performance [34]. These several differences between the two studies caused discrepancies, but further studies are needed to better explain these inconsistent results.

OCD participants showed significantly decreased risk taking on the BART, which suggest that people with OCD avoid taking risks. Risk taking involves the potential for danger or harm as well as the opportunity to obtain some form of reward [19]. The BART scores successfully predicted naturalistic risk taking [48]. namely, the likelihood of engaging in real-world activities that involve potential negative outcomes [49]. Although no previous study has used the BART to investigate OCD, our results are generally consistent with previous research showing lower risk taking or more risk averting properties in people with OCD [50], [51], [52]. OCD participants had lower risk taking than controls in everyday activities, as measured by the self-reported Everyday Risk Inventory, in both American [52] and Australian samples [51]. Recently, Admon et al. [50] found that people with OCD were reluctant to make risky choices during an interactive risky choice game. Risk taking propensity is determined by both increased reward seeking and decreased sensitivity to loss [53]. Several studies reported decreased sensitivity to reward, and increased sensitivity to loss in OCD [54]. Therefore, the lower risk taking among OCD participants in our study might result from increased sensitivity to loss. This finding is also in line with the maladaptive behavior of OCD patients involving excessive aversion to slight risk, which is commonly thought to result from their perception of situations as highly threatening.

We found that the OCD participants showed significantly higher total scores than controls did on the attentional and motor subscale scores but not on the non-planning subscale of the BIS-11. Most previous studies have also consistently reported higher total and attentional subscale scores of the BIS-11 in OCD [37], [55], [56]. In terms of motor subscale of BIS-11, there are some controversies. Contrary to ours, some previous studies could not find any difference of motor impulsiveness of BIS-11 between OCD patients and controls [37], [55], [57]. However, in one recent Korean study, the motor impulsiveness of the BIS-11 in OCD patients was significantly higher than in controls and was correlated with hoarding or aggressive/checking dimensions of obsessive-compulsive symptoms [56]. In another Korean study, although the difference in motor impulsiveness between OCD patients and controls did not reach statistical significance, the effect size (d = 0.81) was bigger than in ours (d = 0.73), which means that the main reason for their being no difference in motor impulsiveness was the small sample size (OCD patients, n = 18; controls, n = 33). Consistent with previous findings, our study showed no difference in non-planning impulsiveness between OCD and control participants [55], [57].

To our knowledge, this is the first study of OCD that used the SST, DDT, and BART altogether. All of the three behavioral measures of impulsivity demonstrated differences between groups. In OCD subjects, the performances of the SST, DDT, and BART were not correlated with each other. This finding suggested that there were no associations between behavioral disinhibition, impulsive decision making, and unduly risk taking. Several investigations that used multiple measures of impulsivity simultaneously in the study of other psychiatric disorders, such as substance use disorders or impulse control disorders, also showed inconsistent profiles between measures (i.e., increased in one dimension but null findings in other dimensions of impulsivity) [17]. In our study, increased behavioral disinhibition and impulsive decision making but decreased risk taking were observed in OCD subjects. Such a result might be possible when each task reflects a distinct underlying process. The null correlations between task parameters are consistent with some prior investigations [58], [59], which also suggest that various assessments of impulsivity are distinct from each other and that there might be different neurobiological mechanisms underlying each process [60]. Although speculative, it is conceivable that each process could contribute to the OCD phenotype in different ways. In the simple case of a patient with pathologic doubt and checking, risk aversion could lead to a greater preference for avoiding risky situations, whereas a concomitant inability to wait for tension relief may provoke safety behaviors (e.g., checking the gas valve), and the inability to stop already started behaviors leads to repeating those behaviors. As aforementioned, our study supports the utility of implementing multiple behavioral tasks for measuring impulsivity because of its conceptual complexity.

One limitation of the present study was that all of the patients were taking SRIs and some of the patient were also taking benzodiazepines and/or atypical antipsychotics when they were tested, which may have had confounding effects on our results. To rule out these potential confounding effects, further research using drug naïve or drug free OCD subjects is warranted. Another limitation is that we may not have completely excluded OCD subjects with comorbid childhood onset psychiatric conditions, especially ADHD. Given that such comorbid conditions were ruled out by a lifetime history–taking method relying solely on patients' self-reports, it may have been possible that some hidden adult ADHD patients were included in both the OCD and healthy control groups. Considering that ADHD tends to be highly comorbid with OCD and impaired response inhibition [61], [62], our results may have at least been partially influenced by unscreened participants with ADHD or other childhood onset psychiatric conditions. The other study limitation is that a portion of participants (n = 20 in OCDs and n = 24 in healthy controls) was excluded because of invalid SST performance. A similar rate of exclusion due to invalid results on the SST has been observed in several other studies [61], [63], [64]. In our study, majority (n = 32) of the outliers were excluded due to high stop response inhibition rate (>70%), which reflects participants' trading speed in the go task for success in the stop task [65], [66]. Other researchers also pointed out that participants are likely to trade-off speed for accuracy, instead of equally considering both aspects of the instructions, that is, fast responses in go trials and inhibition in stop trials [65], [67]. Considering these findings including our outliers, our instruction might not be sufficient for the participants to balance go-process and stop-process. Some researchers suggested several strategies for preventing this trade- off such as keeping the proportion of stop signal as low as possible [68] or providing clear instruction (e.g., by stressing speed in the go task and explaining the staircase-tracking procedure) and implementing feedback procedures (feedback after every trial or every block) [69]. Therefore, future studies with these strategies can minimize the outliers for reliable SST.

In summary, this study showed increased action and choice impulsivity but lower risk taking characteristics in OCD patients who have difficulty in waiting for advantageous outcomes and stopping already started behavior. Our multimodal tasks results support each task as a measure of a distinct underlying process of impulsivity.
